# Retraction Note: Study the effect of static magnetic field intensity on drug delivery by magnetic nanoparticles

**DOI:** 10.1038/s41598-022-18378-3

**Published:** 2022-08-16

**Authors:** Abbas Moghanizadeh, Fakhreddin Ashrafzadeh, Jaleh Varshosaz, Antoine Ferreira

**Affiliations:** 1 Department of Materials Engineering, Isfahan University of Technology, Isfahan, 84156‑83111 Iran; 2 Department of Pharmaceutics, School of Pharmacy and Pharmaceutical Sciences, Isfahan University of Medical Sciences, Isfahan, Iran; 3 INSA Centre Val de Loire, Université d’Orléans, PRISME, EA4229 Bourges, France

Retraction of: *Scientific Reports* 10.1038/s41598-021-97499-7, published online 10 September 2021

The Editors have retracted this Article.

After publication of this Article it was brought to the Editors' attention that some of the data appear to overlap with those in another article from these authors^1^. Specifically:

- the TEM image of Fe3O4 nanoparticles in Figure 1 appears to be identical with the TEM image of zinc ferrite in Figure 3 in^1^;
- the Fe3O4 VSM data in Figure 2B appear to be identical with the VSM data for zinc ferrite in Figure 4B in^1^;
- there are unusual irregularities in the FTIR spectra shown in Figure 3.

The Authors are unable to provide the raw data underlying these experiments; the Editors therefore no longer have confidence in the results presented.

Antoine Ferreira agrees with this retraction and its wording. Fakhreddin Ashrafizadeh disagrees with this retraction. Jaleh Varshosaz did not respond to the correspondence from the Editors about this retraction. Abbas Moghanizadeh has not explicitly stated whether they agree or disagree with this retraction.
